# Impact of Different Positive End-Expiratory Pressures on Lung Mechanics in the Setting of Moderately Elevated Intra-Abdominal Pressure and Acute Lung Injury in a Porcine Model

**DOI:** 10.3390/jcm10020306

**Published:** 2021-01-15

**Authors:** Mascha O. Fiedler, Emilis Simeliunas, B. Luise Deutsch, Dovile Diktanaite, Alexander Harms, Maik Brune, Maximilian Dietrich, Florian Uhle, Markus A. Weigand, Armin Kalenka

**Affiliations:** 1 Department of Anesthesiology, Heidelberg University Hospital, 69120 Heidelberg, Germany; simeliunui@gmail.com (E.S.); diktanaite@gmail.com (D.D.); maximilian.dietrich@med.uni-heidelberg.de (M.D.); florian.uhle@med.uni-heidelberg.de (F.U.); Markus.weigand@med.uni-heidelberg.de (M.A.W.); 2 Translational Lung Research Center Heidelberg (TLRC), German Center for Lung Research (DZL), 69120 Heidelberg, Germany; armin.kalenka@kkh-bergstrasse.de; 3 Department of Anesthesiology, Kantonsspital Lucerne, 6004 Lucerne, Switzerland; 4 Faculty of Medicine, Justus Liebig University, 35392 Giessen, Germany; luisedeutsch@gmx.de; 5 Institute of Pathology, University Hospital Heidelberg, 69120 Heidelberg, Germany; alexander.harms@med.uni-heidelberg.de; 6 Department of Internal Medicine I and Clinical Chemistry, University Hospital Heidelberg, 69120 Heidelberg, Germany; maik.brune@med.uni-heidelberg.de; 7 Department of Anesthesiology and Intensive Care Medicine, Hospital Bergstrasse, 64646 Heppenheim, Germany; 8 Faculty of Medicine, University of Heidelberg, 69120 Heidelberg, Germany

**Keywords:** ALI, ARDS, intraabdominal pressure, PEEP, end-expiratory lung volume, transpulmonary pressure

## Abstract

The effects of a moderately elevated intra-abdominal pressure (IAP) on lung mechanics in acute respiratory distress syndrome (ARDS) have still not been fully analyzed. Moreover, the optimal positive end-expiratory pressure (PEEP) in elevated IAP and ARDS is unclear. In this paper, 18 pigs under general anesthesia received a double hit lung injury. After saline lung lavage and 2 h of injurious mechanical ventilation to induce an acute lung injury (ALI), an intra-abdominal balloon was filled until an IAP of 10 mmHg was generated. Animals were randomly assigned to one of three groups (group A = PEEP 5, B = PEEP 10 and C = PEEP 15 cmH_2_O) and ventilated for 6 h. We measured end-expiratory lung volume (EELV) per kg bodyweight, driving pressure (ΔP), transpulmonary pressure (ΔP_L_), static lung compliance (C_stat_), oxygenation (P/F ratio) and cardiac index (CI). In group A, we found increases in ΔP (22 ± 1 vs. 28 ± 2 cmH_2_O; *p* = 0.006) and ΔP_L_ (16 ± 1 vs. 22 ± 2 cmH_2_O; *p* = 0.007), with no change in EELV/kg (15 ± 1 vs. 14 ± 1 mL/kg) when comparing hours 0 and 6. In group B, there was no change in ΔP (26 ± 2 vs. 25 ± 2 cmH_2_O), ΔP_L_ (19 ± 2 vs. 18 ± 2 cmH_2_O), C_stat_ (21 ± 3 vs. 21 ± 2 cmH_2_O/mL) or EELV/kg (12 ± 2 vs. 13 ± 3 mL/kg). ΔP and ΔP_L_ were significantly lower after 6 h when comparing between group C and A (21 ± 1 vs. 28 ± 2 cmH_2_O; *p* = 0.020) and (14 ± 1 vs. 22 ± 2 cmH_2_O; *p* = 0.013)). The EELV/kg increased over time in group C (13 ± 1 vs. 19 ± 2 mL/kg; *p* = 0.034). The P/F ratio increased in all groups over time. CI decreased in groups B and C. The global lung injury score did not significantly differ between groups (A: 0.25 ± 0.05, B: 0.21 ± 0.02, C: 0.22 ± 0.03). In this model of ALI, elevated IAP, ΔP and ΔP_L_ increased further over time in the group with a PEEP of 5 cmH_2_O applied over 6 h. This was not the case in the groups with a PEEP of 10 and 15 cmH_2_O. Although ΔP and ΔP_L_ were significantly lower after 6 hours in group C compared to group A, we could not show significant differences in histological lung injury score.

## 1. Introduction

The average intra-abdominal pressure (IAP) on admission of ventilated critical care patients in the intensive care unit is around 10 mmHg [1]. The pressure in the abdomen causes a cranial shift of the diaphragm, thereby increasing intra-thoracic pressure and affecting lung volumes and respiratory mechanics [2,3]. The presence of intra-abdominal hypertension (IAH) is associated with a decrease in lung volume [4] and chest wall compliance [5]. An increasing degree of IAP results in a decline in lung volume [6,7], while decompressive laparotomy results in an improvement in lung volume [4]. Patients receiving mechanical ventilation are more likely to have IAH [8,9]. The presence of IAH may add to the development of ventilator-induced lung injury (VILI) [10]. In the setting of IAH, the lung will collapse at higher closing pressures during expiration. In the context of IAH, increased atelectrauma due to increased atelectasis formation and an insufficient positive end-expiratory pressure (PEEP) may further accelerate lung injury [2,3,11].

Nearly every fourth patient requiring mechanical ventilation has acute respiratory distress syndrome (ARDS) [12]. The presence of ARDS appears to strongly influence how IAP affects respiratory mechanics and oxygenation [3].

The best procedure to set PEEP in patients with ARDS is still a matter of debate [13]. Determining the best PEEP to be used during increasing abdominal pressure and ARDS remains unknown and PEEP settings in clinical routines can be even more challenging.

We therefore studied the effect of a moderately elevated IAP of 10 mmHg on lung mechanics in a porcine ALI model up to 6 h. To investigate the consequences of different PEEP levels, we used three levels of PEEP (5, 10, 15 cmH_2_O). The hypothesis in our study was that a PEEP of 15 cmH_2_O in moderately elevated IAP (10 mmHg) would be protective by reducing driving pressure and transpulmonary pressures, as well as preserving the EELV during mechanical ventilation.

## 2. Materials and Methods

### 2.1. Animal Preparation and Instrumentation

The protocol was approved by the responsible committee for animal research (Regierungspräsidium Karlsruhe, No. 35-9185.81/G-161/17). We included 18 female German landracer swines in this study with a weight of 50 ± 3 kg. After induction of anesthesia, the pigs were ventilated with an intensive care ventilator (Carescape R860, GE Healthcare, Madison, USA) using an inspiratory oxygen concentration (F_i_O_2_) of 0.4 in a pressure-controlled mode with volume guaranty. Also, a tidal volume of 8 mL/kg bodyweight (bw), an inspiration/expiration ratio (I:E) of 1:2 and a PEEP of 5 cmH_2_O was provided. The respiratory rate was adjusted to p_a_CO_2_ (normocapnia). Anesthesia was maintained by continuous infusion of 6 mg/kg/h Ketanest S (Pfizer Pharma, Berlin, Germany), 3.6 mg/kg/h midazolam and 10–30 mg/kg/h propofol 2% (Propofol, Fresenius Kabi, Bad Homburg, Germany). There was no use of neuromuscular blockers. Adequacy of the depth of anesthesia was regularly assessed by the absence of spontaneous breathing efforts and lack of muscle tone. No recruitment maneuvers were applied through the study period. Throughout the whole experiment, pigs were kept in a supine position.

A 5 French catheter was inserted with ultrasound guidance (VScan^®^, GE Ultrasound, Horten, Norway) in the femoral artery for measurement of invasive blood pressure and cardiac index (CI) (PiCCO^®^, Pulsion Medical systems, Feldkirchen, Germany). A three-lumen catheter (Logicath^®^, Smiths Medical, Grasbrunn, Germany) was inserted with ultrasound guidance in the right external jugular vein. Crystalloid solution (Sterofundin^®^, Braun, Melsungen, Germany) was infused at a rate of 10 mL/kg for the first hour, and thereafter the infusion rate was kept at 10–40 mL/kg/h to maintain hemodynamic stability during the experiment. A polyethylene catheter with a thin walled latex balloon (Nutrivent multifunction nasogastric catheter, Sidam, San Glacomo Roncole, Italy) was passed via the snout into the stomach. The catheter was connected to the pressure-port of the ventilator for measuring esophageal pressure (P_Es_). After inflating the balloon with 3 mL of air, it was withdrawn into the esophagus. Appropriate catheter position was confirmed by visualization of cardiac artefacts on the P_Es_ curve on the ventilator and further confirmed by an occlusion test [14].

After a midline laparotomy, a large intra-abdominal balloon (200 L weather balloon, Stratoflight, Blomberg, Germany) was placed in the peritoneal cavity. Correct position in all abdominal quadrants was ensured by visual inspection and partial inflation. The abdomen was carefully closed. A urine catheter was placed in the bladder and connected to an intra-abdominal pressure device (UnoMeter^®^ Abdo-Pressure, Birkerod, Denmark).

Acute lung injury was established by using 0.9% sodium chloride warmed to body temperature, instilled into the endotracheal tube, and then drained by gravity [15]. The animals remained in a supine position and saline lung lavage was repeatedly performed until a ratio of partial arterial pressure of oxygen to inspired oxygen (P/F ratio) <150 mmHg was reached for at least 30 min. Thereafter, an injurious mechanical ventilation was applied (pressure controlled ventilation: peak inspiratory airway pressure (P_Insp_): 35 cmH_2_O, PEEP: 0 cmH_2_O, respiratory rate (RR): 12/minute, I:E: 1:2 and F_i_O_2_: 1.0 for 120 min.

### 2.2. Measurements and Calculations

Peak inspiratory airway pressure, PEEP, inspiratory esophageal pressure (P_EsInsp_) and end-expiratory esophageal pressure (P_EsExp_) were recorded from the ventilator. ΔP and ΔP_L_ were calculated as previously described [16]. Transpulmonary inspiratory pressure (TPP_Insp_) was calculated as TTP_Insp_ = P_Insp_ − P_EsInsp_ and transpulmonary expiratory pressure (TPP_Exp_) as TPP_Exp_ = PEEP − P_EsExp_. C_Stat_ was measured by the ventilator during an inspiratory hold. Elastance of the respiratory system (E_RS_) was calculated as E_RS_ = (P_Insp_ − PEEP)/V_T_, chest wall elastance (E_CW_) as E_CW_ = (P_EsInsp_ − P_EsExp_)/V_T_ and elastance of the lung (E_L_) as E_L_ = E_RS_ − E_CW_.

We measured EELV bedside without interruption of mechanical ventilation using the modified nitrogen multiple breath (NMBW) technique [17], which is integrated in the intensive care ventilator. We performed 3 wash-out/wash-in cycles and averaged them within a 10% range. Cardiac index (CI) was calculated with the PiCCO^®^ System. End-expiratory IAP (IAP_Endex_) was measured as recommended [18,19] and zeroed at the midaxillary level [20]. P/F ratio was calculated based on the ratio of partial arterial pressure of oxygen to F_i_O_2_.

### 2.3. Experimental Protocol

After initial instrumentation, the pigs were stabilized for 30 min and baseline measurements were taken. After induction of acute lung injury by saline lung lavage and 120 min injurious mechanical ventilation, we changed ventilator parameters to baseline settings and measured values for hour 0 (H0).

**Figure 1:**
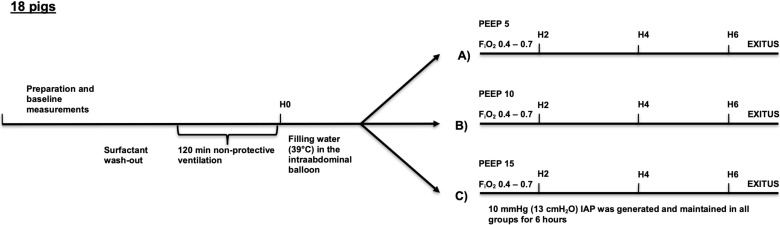
Experimental timeline. H0, H2, H4, H6—hours after lung injury (wash out and injurious ventilation), PEEP—positive end expiratory pressure, FiO_2_—oxygen fraction, IAP—intra-abdominal pressure, (**A**)—group A, (**B**)—group B, (**C**)—group C.

The abdominal balloon was then filled with water up to an IAP_Endex_ of 10 mmHg. The animals were then randomized into group A (*n* = 6) with a PEEP of 5 cmH_2_O, group B (*n* = 6) with a PEEP of 10 cmH_2_O, or group C (*n* = 6) with a PEEP of 15 cmH_2_O for 6 h (H6) (Figure 1). The F_i_O_2_ was kept between 0.4 and 0.7 to reach a saturation higher than 85%.

At the end of the experimental protocol, the pigs were euthanized. We exposed the complete right lung and regional lung samples for extraction to evaluate the wet-dry weight ratio and to perform histological examinations.

### 2.4. Histology

Samples from the anterior, medial and dorsal position of the medial lobe were selected and immediately fixed in formalin. After fixation, the tissue samples were dehydrated and embedded. The sections were stained with hematoxylin and eosin. A pathologist, blinded to the study variables, evaluated each sample histologically to determine a lung injury score. To quantify the extent of histologic lung injury, the pathologist used a lung injury scoring system [21] (Supplementary Figure S1). Five independent variables were scored to generate the lung injury score. The sum of each of the five independent variables were weighted according to the relevance for acute lung injury [21]. The resulting lung injury score ranges from 0 to 1. Zero represents minimal to no damage and 1 represents the worst damage possible (Supplementary Figure S2).

### 2.5. Wet-To-Dry Ratio

Wet-dry weight ratio was measured in samples from the medial lobe. Samples were weighted, dried and then weighted again. We dried the lung samples for 72 h in an oven at 80 °C.

### 2.6. Statistical Analysis

Sample size was calculated based on expected alterations in end-expiratory lung volume (EELV) from data from previous studies [22] and unpublished data in our lab between a PEEP level of 5 and 10 cmH_2_O. To identify a significant difference in EELV based on an alpha = 0.05 and a power = 80%, a sample size of *n* = 6 per group was calculated to be sufficient. We used the free software G*Power 3 to calculate the sample size [23].

Statistical analysis was performed using SPSS (version 25). Baseline data and H0 values were analyzed with the Shapiro-Wilk test for normal distribution. In case of normally distributed data, a one-way ANOVA was performed. In case of significance, a post hoc analysis with a Games–Howell correction for multiple tests was performed. We used a paired sample t-test to compare baseline with H0 data and H0 with H6 data within one group. Non-normally distributed data were analyzed using non-parametric tests.

To compare H0 with H6, data between all groups were used to calculate the difference between H6 and H0, followed by one-way ANOVA. In case of significance, post-hoc analyses with a Games–Howell correction for multiple tests were performed.

To demonstrate the impact of the different PEEP levels over six hours of ventilation, we generated the pulmonary and hemodynamic parameters by the subtraction of H0 from H6. A positive result was interpreted as an increase in this parameter.

Data are expressed as mean ± standard error of the mean (SEM) in cases of non-normal distribution, otherwise as median and interquartile range (IQR). For all tests, *p* < 0.05 was considered statistically significant.

## 3. Results

After induction of acute lung injury at H0, there were several significant changes in values compared with baseline data (Table 1): a reduction in EELV(1269 ± 68 vs. 665 ± 54; *p* < 0.010), C_stat_ (42 ± 2 vs. 21 ± 1; *p* < 0.013) and P/F ratio (456 ± 13 vs. 110 ± 13; *p* < 0.012) were seen, as well as an increase in ∆P (12 ± 0 vs. 26 ± 1; *p* < 0.009) and ∆P_L_ (6 ± 0 vs. 19 ± 1; *p* < 0.011).

**Figure 2:**
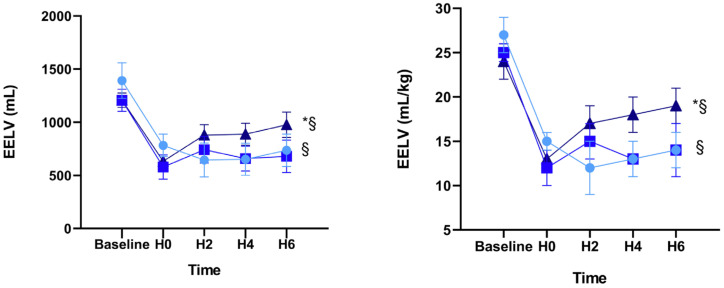
Alterations of end-expiratory lung volume in absolute values and in relation to body weight in response to acute lung injury and intra-abdominal pressure of 10 mmHg over 6 h of mechanical ventilation. ● = group A with PEEP: 5 cmH_2_O, ■ = group B with PEEP: 10 cmH_2_O, ▲ = group C with PEEP: 15 cmH_2_O. * = *p* < 0.05 H0 vs. H6; § = *p* < 0.05 group A vs. group C.

**Figure 3:**
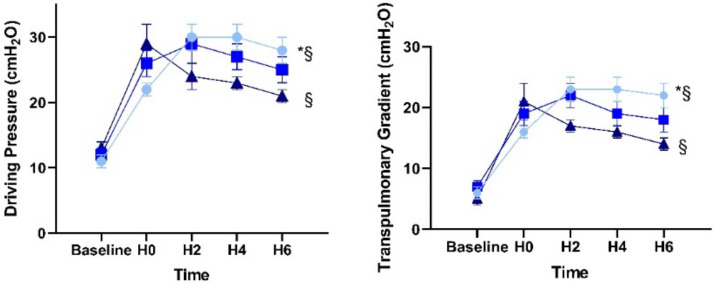
Alterations of driving pressure and transpulmonary gradient in response to acute lung injury and intra-abdominal pressure of 10 mmHg over 6 h of mechanical ventilation. ● = group A with PEEP: 5 cmH_2_O, ■ = group B with PEEP: 10 cmH_2_O, ▲ = group C with PEEP: 15 cmH_2_O. * = *p* < 0.05 H0 vs. H6 § = *p* < 0.05 group A vs. group C.

When compared with data at H0, we observed several significant alterations in the setting of 6 h of mechanical ventilation with an intra-abdominal pressure of 10 mmHg and acute lung injury (H6). The EELV and EELV/kg increased in group C (EELV: 633 ± 39 vs. 976 ± 119 mL; *p* = 0.029 and EELV/kg bw: 13 ± 1 vs. 19 ± 2 mL/kg; *p* = 0.034) but did not change in groups A and B (Table 2 and Figure 2). The ∆P increased in group A over time (22 ± 1 vs. 28 ± 2; *p* = 0.006) whereas it decreased (without significance) with a PEEP of 15 cmH_2_O in group C (29 ± 3 vs. 21 ± 1; *p* = 0.081) and showed significantly lower values than group A at H6 (C: 21±1 vs A: 28 ± 2; *p* = 0.020) (Table 2 and Figure 3).

The CI was lower in groups B and C between H0 and H6 (4.4 ± 0.3 vs. 3.9 ± 0.2; *p* = 0.038 and 5.2 ± 0.4 vs. 3.7 ± 0.1; *p* = 0.018). Group C received more fluids (mL) than group A (7125 ± 573 vs. 4900 ± 433; *p* = 0.030). Despite alterations in the CI, the P/F ratio increased in all groups after 6 h.

The calculated difference in EELV values at H6 and H0 (EELV_H6 – H0_) showed the increase of EELV_H6 – H0_ at PEEP 15 cmH_2_O (group C) and the decrease at PEEP 5 cmH_2_O (group A) (group C EELV_H6 – H0_ = 343 ± 113 vs. group A: EELV_H6 – H0_ = −46 ± 115, *p* = 0.010) and this also related to body weight (kg) (group C: EELV/kg_H6 – H0_ = 7 ± 2 vs. group A: EELV/kg_H6 – H0_ = −1 ± 2, *p* = 0.015) (Table 3). All animals completed the study.

### Lung Injury Score and Wet-Dry Weight Ratio (W/D)

The global lung injury score did not differ between the groups (A: 0.25 ± 0.05, B: 0.21 ± 0.02, C: 0.22 ± 0.03; *p* = 0.520) (Supplementary Figure S1).

The wet-dry weight ratios (W/D) also did not differ between groups (A: 1.64 ± 0.27, B: 1.98 ± 0.46, C: 1.95 ± 0.29; *p* = 0.391).

## 4. Discussion

### 4.1. Main Findings

The present animal study demonstrated that a PEEP of 15 cmH_2_O in cases of moderately elevated IAP seems to improve oxygenation by increasing the EELV without aggravating the lung injury in piglets with saline lavage-induced ALI.

### 4.2. Lung Injury Model and Elevated Intra-Abdominal Pressure

Our study involved the use of a triple hit porcine model. Up to 25% of mechanical ventilated patients on an intensive care unit (ICU) have ARDS [12]. This acute lung injury model was established by repeated saline lung lavages and non-protective ventilation—the classical model used to simulate human ARDS in an animal [21,24]. The third hit was a moderately elevated IAP, established by using an intra-abdominal balloon filled with water. This model should be representative of critically ill patients in an ICU as the mean IAP in these patients is around 10 mmHg [1]. We thus tried to simulate a well-known situation in critically ill patients. Lima et al. found that a 3 h exposure to an IAP of 15 mmHg was sufficient to cause alveolar collapse, hemorrhage, interstitial edema, and neutrophil infiltration in the lungs and increased lung cell apoptosis despite the application of lung-protective ventilation in a study of 20 rats [10]. Our group recently showed that an IAP of 10 mmHg in a porcine study over 6 h caused lung injury which can be reduced by the application of PEEP [22].

In 2012, Regli et al. [7] carried out a porcine study with IAH and ALI in a different setting. They induced ALI by oleic acid and randomly applied three different IAP (3, 18 and 22 mmHg) by filling an intra-abdominal balloon with air to study the effects of different PEEP settings (baseline PEEP (5 cmH_2_O), moderate PEEP (0.5 × IAP in cmH_2_O) and high PEEP (1.0 × IAP in cmH_2_O)) in short time alterations [7]. Their study supported the application of PEEP in the setting of acute lung injury and IAH matched to the IAP in short time experiments. We filled the balloon with warm water. The effect of this on the cephalad shift of the diaphragm was, from our point of view, a more pathophysiological approach (e.g., ascites, abdominal distension, fluid overload) [3]. There are limited data to show at what level of IAP lung volumes reduce or atelectasis occurs. Regli et al. showed in different studies that at least in pigs, lung volumes decline with an increasing degree of IAH [6,7]. The experimental setting of Mutoh et al. in piglets involved the inflation of an abdominal balloon in small increments and they found that end-expiratory lung volumes (EELV) reduced even after small increases in IAP (ranged from 13, 14, 15 to finally 16 cmH_2_O) [25]. In our model, we were able to show that even a moderate IAP of 10 mmHg caused changes in alveolar mechanics when applied over 6 h [22].

In our study, the P/F ratio improved over the 6 h of study protocol after the triple-hit model was established in all groups. The difference over time was more profound with increasing PEEP. Interestingly, it looks like IAH has a severe effect on oxygenation only in injured lungs [7,26]. Oxygenation, as a target for PEEP optimization during mechanical ventilation, has become widely accepted in clinical routine. Thus, the ARDS network table, which contains relatively fixed combinations of F_i_O_2_ and PEEP, is commonly used at the bedside, although individual lung mechanics are not considered [27]. For instance, atelectrauma could not be identified by oxygenation in an experimental model of acute lung injury [28].

### 4.3. Alterations in Lung Mechanics

The main aim of our study was to describe the alterations in EELV and ΔP_L_ in an ALI porcine model of moderately elevated IAP. Stress (the transpulmonary pressure (ΔP_L_)) and strain (the applied tidal volume per end-expiratory lung volume (V_T_/EELV)) are crucial parameters for the development and prevention of ventilator-induced lung injury (VILI) [29,30]. Unfortunately, neither parameter is routinely measured at the bedside [12]. We detected a further drop of EELV and EELV/kg bw with a PEEP of 5 cmH_2_O over time in our study. In the two groups with a PEEP of 10 and 15 cmH_2_O, these reductions were not observed. The experimental study of Steinberg et al. showed that PEEP converted abnormal, unstable alveoli into normal functioning, stable alveoli and PEEP-induced alveolar stabilization reduced lung damage [31]. An increase in PEEP may have caused progressive distention in the conducting airways rather than increasing alveolar area and stability [28]. Measuring EELV might be an attractive method to adjust PEEP appropriately for patients in ICU.

After the induction of the triple-hit model, our results showed increased values in ΔP_L_ as well as in ΔP, a surrogate for ΔP_L_. Obviously, during six hours of mechanical ventilation, ΔP_L_ as well as ΔP decreased with a PEEP of 15 cmH_2_O, while the opposite was seen in the group with PEEP of 5 cmH_2_O. As ΔP is a predictor of mortality in ARDS patients, a reduction of ΔP and ΔP_L_ by PEEP seems to be a lung protective approach [32].

Previous investigations confirmed an increase in peak and plateau airway pressures proportionally with increasing IAP [33,34]. In a pig model of IAH, increasing intra-abdominal volume has also been shown to exponentially increase peak airway pressure [35]. In injured porcine lungs with elevated IAP, the E_RS_ is not only influenced by alterations of the E_CW_, but also by alterations in E_L_. Our study demonstrated that at the end of a 6 h mechanical ventilation, E_RS_ decreased with increasing PEEP mainly due to the decrease in E_L_ with raising PEEP, but E_CW_ was nearly the same in all groups. Similar results were shown in patients. Krebs et al. applied different PEEP levels (up to 20 cmH_2_O) in 20 patients with ARDS. One half of the study population had IAH (with a mean IAP of 8 and 16 mmHg, respectively) [36]. PEEP was found to decrease E_RS_ by decreasing E_L_ without influencing E_CW_ in both groups.

### 4.4. Limitations

This is an animal study with an artificially induced lung injury. The results therefore cannot be applied to human patients without any restrictions. We only used an IAP of 10 mmHg and PEEP of 5, 10 and 15 cmH_2_O. Hence, it is unclear to what extent the above-mentioned PEEP values matching to IAP is useful and would be tolerated at higher IAP values. Despite lower values for stress and strain, we could not find differences in histological lung injury score (Supplementary Figure S1). This might be due to a short ventilation time or mild differences between the groups.

It is still not clear if the PEEP strategies as used in our study affected acute lung injury. This is because the changes in lung mechanics and gas exchange were not accompanied by changes in the lung injury score or W/D. This discrepancy could perhaps be due to the low sensitivity of injury scores and W/D, which sometimes shows no change when other markers of inflammation and permeability disruption such as broncho-alveolar-lavage (BAL) cellularity or high molecular weight protein concentrations reach significance. Alternatively, it could be due to the lack of certain controls (W/D) and scores in pigs at H0. Perhaps the oxygenation and lung mechanic changes were unrelated to the actual injury, and instead due to variations in the severity of micro-atelectasis or other factors.

## 5. Conclusions

We studied different PEEP levels in a model of ALI and elevated IAP. In the group with a PEEP of 5 cmH_2_O applied over 6 h, ΔP and ΔP_L_ increased further over time. This was not the case in the groups with a PEEP of 10 and 15 cmH_2_O. Only in the group with a PEEP of 15 cmH_2_O, the EELV and EELV/kg bw increased significantly over time. Although ΔP and ΔP_L_ were significantly lower at H6 in group C compared to group A, we could not show significant differences in histological lung injury score nor in the wet/dry ratio of the lungs between the groups.

## Figures and Tables

**Table 1:** Pulmonary and hemodynamic parameters after induction of acute lung injury at H0 compared with baseline data.

	Baseline	H0
IAP	2.3 ± 0.3	3.2 ± 0.3 *
EELV	1269 ± 68	665 ± 54 *
EELV/kg	25 ± 1	13 ± 1 *
∆P	12 ± 0	26 ± 1 *
∆P_L_	6 ± 0	19 ± 1 *
TPP_Insp_	6 ± 1	17 ± 1 *
TPP_Exp_	0 ± 0	−2 ± 1 *
C_stat_	42 ± 2	21 ± 1 *
E_RS_	30 ± 1	65 ± 4 *
E_CW_	15 ± 1	18 ± 1 *
E_L_	15 ± 1	47 ± 4 *
HR	66 ± 3	83 ± 5 *
MAP	84 ± 2	101 ± 2 *
P/F ratio	456 ± 13	110 ± 13 *
CI	4.2 ± 0.2	4.7 ± 0.2
Lactate	1.4 ± 0.2	1.4 ± 0.1
pH	7.45 ± 0.01	7.37 ± 0.01
RR	20 (0)	24 (4)

*n* = 18, data are expressed as mean ± standard error of the mean (SEM) with the exception of respiratory rate (RR) which is expressed as median (IQR). IAP = intra-abdominal pressure (mmHg), EELV = end-expiratory lung volume (mL), EELV/kg = end-expiratory lung volume per kg bodyweight (ml/kg), ∆P = driving pressure (cmH_2_O), ∆P_L_ = transpulmonary pressure (cmH_2_O), TPP_Insp_ = inspiratory transpulmonary pressure (cmH_2_O), TPP_Exp_ = expiratory transpulmonary pressure (cmH_2_O), C_Stat_ = static pulmonary compliance (mL/cmH_2_O), E_RS_ = elastance of the respiratory system (cmH_2_O/mL), E_CW_ = elastance of the chest wall (cmH_2_O/mL), E_L_ = lung elastance (cmH_2_O/mL), HR = heart rate (beats/min), MAP = mean arterial pressure (mmHg), P/F ratio = ratio between arterial pressure of oxygen and inspired oxygen concentration (mmHg), CI = cardiac index (L/min/m^2^), Lactate (mmol/L). * *p* < 0.05.

**Table 2:** Pulmonary and hemodynamic parameters in the setting of 6 h of mechanical ventilation with an intra-abdominal pressure of 10 mmHg and acute lung injury.

		Group A (*n* = 6)	Group B (*n* = 6)	Group C (*n* = 6)
Weight (kg)	Baseline	51 ± 3	49 ± 3	51 ± 3
PEEP		5	10	15
IAP	H0	3 ± 1	3 ± 0	4 ± 1
IAP	H6	10 ± 1 *	10 ± 0 *	10 ± 0 *
EELV	H0	782 ± 105	579 ± 114	633 ± 39
EELV	H6	736 ± 152	680 ± 153	976 ± 119 *
EELV/kg	H0	15 ± 1	12 ± 2	13 ± 1
EELV/kg	H6	14 ± 2	13 ± 3	19 ± 2 *
∆P	H0	22 ± 1	26 ± 2	29 ± 3
∆P	H6	28 ± 2 *^§^	25 ± 2	21 ± 1
∆P_L_	H0	16 ± 1	19 ± 2	21 ± 3
∆P_L_	H6	22 ± 2 *^§^	18 ± 2	14 ± 1
TPP_Insp_	H0	15 ± 1	17 ± 3	20 ± 2
TPP_Insp_	H6	19 ± 2 *	18 ± 2	18 ± 1
TPP_Exp_	H0	−2 ± 0	−3 ± 1	−1 ± 1
TPP_Exp_	H6	−2 ± 1^§^	−1 ± 0^$^	3 ± 1 *
C_stat_	H0	22 ± 1	21 ± 3	19 ± 2
C_stat_	H6	18 ± 1 *^§^	21 ± 2	24 ± 2 *
E_RS_	H0	56 ± 3	67 ± 7	72 ± 9
E_RS_	H6	69 ± 5 *^§^	66 ± 7	53 ± 5
E_CW_	H0	16 ± 2	17 ± 2	21 ± 2
E_CW_	H6	16 ± 2	18 ± 1	17 ± 2 *
E_L_	H0	40 ± 2	50 ± 7	52 ± 8
E_L_	H6	54 ± 5 *^§^	48 ± 7	35 ± 2
HR	H0	70 ± 8	84 ± 11	93 ± 7
HR	H6	88 ± 6	84 ± 9	75 ± 4
MAP	H0	101 ± 4	99 ± 2	104 ± 6
MAP	H6	108 ± 5	99 ± 5	97 ± 3
P/F ratio	H0	130 ± 22	120 ± 27	81 ± 19
P/F ratio	H6	196 ± 39 *	240 ± 55 *	320 ± 56 *
p_a_CO_2_	H0	38 ± 2	40 ± 2	39 ± 2
p_a_CO_2_	H6	42 ± 1	39 ± 1	38 ± 1
CI	H0	4.5 ± 0.4	4.4 ± 0.3	5.2 ± 0.4
CI	H6	4.2 ± 0.1 ^§^	3.9 ± 0.2 *	3.7 ± 0.1 *
Lactate	H0	1.3 ± 0.3	1.3 ± 0.2	1.6 ± 0.3
Lactate	H6	0.6 ± 0.1	0.6 ± 0.1 *	0.8 ± 0.1
pH	H0	7.38 ± 0.02	7.36 ± 0.01	7.38 ± 0.02
pH	H6	7.44 ± 0.04	7.44 ± 0.01	7.45 ± 0.01
RR	H0	23 ± 1	23 ± 1	25 ± 1
RR	H6	25 ± 1 *	25 ± 1	25 ± 1
Crystalloid volume	H6	4.9 ± 0.4 ^§^	5.9 ± 0.6	7.1 ± 0.6

Data are expressed as mean ± SEM. PEEP = positive end-expiratory pressure (cmH_2_O), IAP = intra-abdominal pressure (mmHg), EELV = end-expiratory lung volume (mL), EELV/kg = end-expiratory lung volume per kg bodyweight (mL/kg), ∆P = driving pressure (cmH_2_O), ∆P_L_ = transpulmonary pressure (cmH_2_O), TPP_Insp_ = inspiratory transpulmonary pressure (cmH_2_O), TPP_Exp_ = expiratory transpulmonary pressure (cmH_2_O), C_Stat_ = static pulmonary compliance (mL/cmH_2_O), E_RS_ = elastance of the respiratory system (cmH_2_O/mL), E_CW_ = elastance of the chest wall (cmH_2_O/mL), EL = lung elastance (cmH_2_O/mL), HR = heart rate (beats/min), MAP = mean arterial pressure (mmHg), P/F ratio = ratio between arterial pressure of oxygen and inspired oxygen concentration (mmHg), p_a_CO_2_ = arterial partial pressure of carbon dioxide, CI = cardiac index (L/min/m^2^), lactate (mmol/L), RR = respiratory rate (1/min). * = *p* < 0.05 H0 vs. H6; § = *p* < 0.05 group A vs. group C; $ = *p* < 0.05 group B vs. group C.

**Table 3:** Pulmonary and hemodynamic parameters generated by the subtraction of H0 from H6 showing the impact of different PEEP levels over 6 h.

	Group A	Group B	Group C
IAP _H6 – H0_	7 ± 1	7 ± 0	7 ± 1
EELV_H6 – H0_	−46 ± 115 ^§^	101 ± 46	343 ± 113
EELV/kg_H6 – H0_	−1 ± 2 ^§^	2 ± 1	7 ± 2
∆P_H6 – H0_	5 ± 1 ^§^	0 ± 1	−8 ± 4
∆P_L H6 – H0_	5 ± 1 ^§^	0 ± 2	−6 ± 3
TPP_Insp H6 – H0_	5 ± 2	1 ± 2	−2 ± 2
TPP_Exp H6 – H0_	−1 ± 1 ^§^	2 ± 1	4 ± 1
C_stat H6 – H0_	−4 ± 0 ^§^	0 ± 1 ^$^	6 ± 2
E_RS H6 – H0_	13 ± 3 ^§^	−1 ± 4	−20 ± 9
E_CW H6 – H0_	0 ± 1	1 ± 1 ^$^	−4 ± 1
E_L H6 – H0_	13 ± 3 ^§^	−2 ± 4	−16 ± 9
HR_H6 – H0_	17 ± 7	0 ± 11	−19 ± 11
MAP_H6 – H0_	7 ± 7	1 ± 5	−7 ± 8
P/F ratio_H6 – H0_	66 ± 21 ^§^	120 ± 35	239 ± 47
CI_H6 – H0_	−0.3 ± 0.4 ^§^	−0.4 ± 0.2	−1.5 ± 0.4
Lactate_H6 – H0_	−0.7 ± 0.3	−0.7 ± 0.1	−0.8 ± 0.3

Data are expressed as mean ± SEM. IAP = intra-abdominal pressure (mmHg), EELV = end-expiratory lung volume (mL), EELV/kg = end-expiratory lung volume per kg bodyweight (mL/kg), ∆P = driving pressure (cmH_2_O), ∆P_L_ = transpulmonary pressure (cmH_2_O), TPP_Insp_ = inspiratory transpulmonary pressure (cmH_2_O), TPP_Exp_ = expiratory transpulmonary pressure (cmH_2_O), C_Stat_ = static pulmonary compliance (mL/cmH_2_O), E_RS_ = elastance of the respiratory system (cmH_2_O/mL), E_CW_ = elastance of the chest wall (cmH_2_O/mL), EL = lung elastance (cmH_2_O/mL), HR = heart rate (beats/min), MAP = mean arterial pressure (mmHg), P/F ratio = ratio between arterial pressure of oxygen and inspired oxygen concentration (mmHg), CI = cardiac index (L/min/m^2^), lactate (mmol/L). § = *p* < 0.05 group A vs. group C. $ = *p* < 0.05 group B vs. group C.

## Data Availability

The data presented in this study are available on request from the corresponding author.

## Supplementary Materials

The following are available online at https://www.mdpi.com/2077-0383/10/2/306/s1, Figure S1: Histologic assessment of lung injury, Figure S2: The Lung Injury Scoring System.

## Abbreviations

ALI	Acute lung injury
ARDS	Acute Respiratory Distress Syndrome
BAL	Broncho-alveolar-lavage
CI	Cardiac index
C_stat_	Static lung compliance
EELV	End-expiratory lung volume
E_L_	Elastance of the lung
E_RS_	Elastance of the respiratory system
HR	Heart rate
IAH	Intra-abdominal hypertension
IAP	Intra-abdominal pressure
IQR	Interquartile range
MAP	Mean arterial pressure
ΔP	Driving pressure
ΔP_L_	Transpulmonary pressure
P_Es_	Esophageal pressure
PEEP	Positive end-expiratory pressure
P_EsInsp_	Inspiratory esophageal pressure
P_EsExp_	End-expiratory esophageal pressure
P/F ratio	PaO_2_/FiO_2_ ratio
RR	Respiratory rate
TPP_Exp_	Transpulmonary expiratory pressure
TPP_Insp_	Transpulmonary inspiratory pressure
VILI	Ventilator-induced lung injury
W/D	Wet-to-dry ratio
