# Supplementary material for: Impact of Different Positive End-Expiratory Pressures on Lung Mechanics in the Setting of Moderately Elevated Intra-Abdominal Pressure and Acute Lung Injury in a Porcine Model
Source: J Clin Med. 2021 Jan 15;10(2):306. doi: 10.3390/jcm10020306 (PMC7830768; doi:10.3390/jcm10020306)
Supplement: Supplementary file 1 [file jcm-10-00306-s001.pdf]

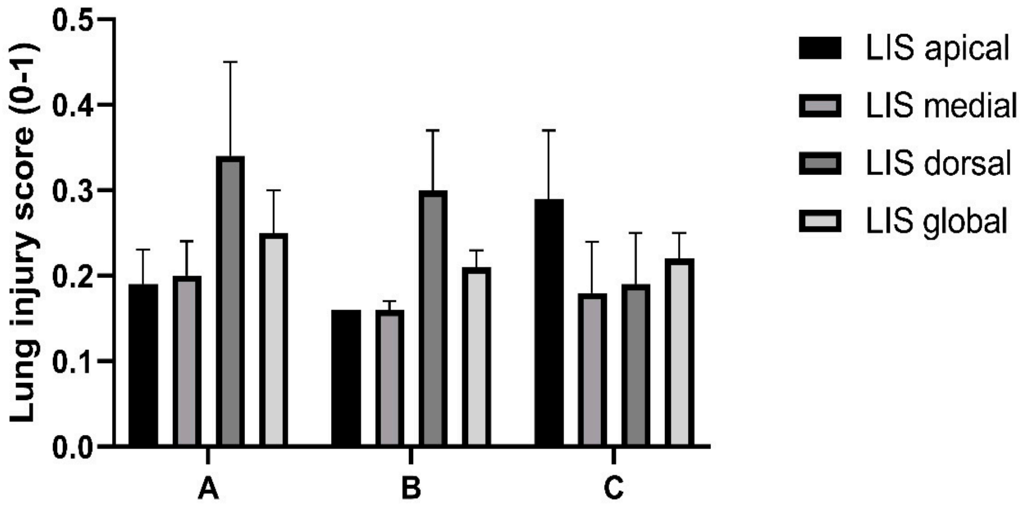

Figure S1. Histologic assessment of lung injury. Quantitative score for lung injury (from 0 = no damage to 1 = maximal alteration) calculated by the averaging score for five independent variables: neutrophils in the alveolar space, neutrophils in the interstitial space, hyaline membranes, proteinaceous debris filling the airspaces and alveolar septal thickening. Apical, medial, dorsal and global score (mean of scores for apical, medial and dorsal) are illustrated.

	Score per field		
**Parameter**	**0**	**1**	**2**
A. Neutrophils in the alveolar space	none	1–5	>5
B. Neutrophils in the interstitial space	none	1–5	>5
C. Hyaline membranes	none	1	>1
D. Proteinaceous debris filling the airspaces	none	1	>1
E. Alveolar septal thickening	<2x	2x–4x	>4x

Score = [(20 × A) + (14 × B) + (7 × C) + (7 × D) + (2 × E)]/(number of fields × 100)

Figure S2: The Lung Injury Scoring System.
